# ALERT-LDL: adherence to guidelines in the treatment of patients with dyslipidemia

**DOI:** 10.1007/s11739-021-02809-6

**Published:** 2021-07-24

**Authors:** Giorgio Bosso, Mariarosaria De Luca, Giovanni Alma, Vincenzo Carbone, Ferdinando Ferrara, Biagio Fimiani, Franco Guarnaccia, Alessandro Iandolo, Sabato Murolo, Maurizio Olivares, Emanuele Romeo, Giosuè Santoro, Antonio Valvano, Giovanni Zito, Ugo Oliviero

**Affiliations:** 1 ARCA (Associazioni Regionali Cardiologi Ambulatoriali), Campania, Italy; 2 Department of Translational Medical Sciences, University Federico II, Via Pansini, 5, 80131 Naples, Italy

**Keywords:** Dyslipidaemia, Cholesterol, Guidelines, Adherence, Targets, Statins

## Abstract

The association between LDL-c levels and cardiovascular outcomes suggests tailoring lipid-lowering therapies according to total cardiovascular risk. We aimed to evaluate the adherence to guidelines-oriented dyslipidaemia’s treatment in an outpatient population referring to ARCA cardiologists, and assess the efficacy of treatment’s optimization for each specific level of risk. Three thousand seventy-five patients enrolled in this prospective study were classified according to cardiovascular risk category, and their therapies were optimized. At the beginning and the 3 month follow-up visit, LDL-c data were collected, and further therapies were prescribed to the patients that did not reach the target. A significant LDL-c reduction was observed in all subgroups at different cardiovascular risk at the end of the study (*p* < 0.05). The number of patients assuming statins, both in monotherapy and in combination with ezetimibe, increased during the follow-up (63% at the enrollment vs 89% after 12 months). At the enrollment, only 1.4% of patients were treated with PCSK-9 inhibitors while after 12 months the percentage increased both in high (5.8%) and very high-risk (18.4%) patients. At the beginning of the study, only 698/3075 patients (22.7%) reached lipid targets. At the end of the study, carried out by the referring cardiologists in the pertaining healthcare districts and specifically aimed to control the lipid profile, the percentage of patients on target increased in all risk categories (68.5%). Our results suggest carefully implementing measures that encourage outpatients and their cardiologists to achieve the targeted lipid profile according to cardiovascular risk.

## Introduction

Dyslipidaemia represents a relevant and preventable component of cardiovascular burden and the serum levels of cholesterol are considered one of the most useful parameters to define cardiovascular risk in adults [1–3]. In particular, several trials have demonstrated a strong relationship between cholesterol levels and the incidence of coronary artery disease [4], as well as between the time of exposure to high values of LDL cholesterol (LDL-c) and cardiovascular risk [5, 6].

Due to the evidence on the effectiveness of lowering lipid levels in improving cardiovascular outcomes [7–10], a relevant interest is growing in lowering cholesterol levels and identifying individuals who could benefit from cholesterol-lowering interventions. Among them, very high-risk patients should achieve an absolute LDL-c goal of < 55 mg/dl, while high-risk patients should aim for a target of < 70 mg/dl. Guidelines did not identify specific goals for HDL-C or triglycerides levels but suggest clinical judgment and particular consideration to these parameters when hypolipidemic treatments are instituted [1].

Despite the well-known dyslipidaemia’s cardiovascular effects and the large prescription of antidyslipidaemic drugs, there are some concerns regarding the rigorous adherence to the guidelines’ suggested lipid targets in real-world practice [11, 12].

ALERT-LDL was a prospective study designed to evaluate the adherence to guidelines-oriented dyslipidaemia’s treatment in an outpatient population referring to ARCA (Associazioni Regionali Cardiologi Ambulatoriali) cardiologists, and to assess the efficacy of the treatment’s optimization for each specific level of risk.

## Materials and methods

### Study protocol

Among the 3648 outpatients consecutively referred to 12 ARCA cardiologists of the pertaining healthcare districts from March 2019 to May 2019, 3075 patients (1602 female/1473 male) were included and completed the study. Thirteen patients, firstly scheduled, died during the observation period, did not complete the program, and were excluded from the analysis.

The inclusion criteria were age between 45 and 75 years and diagnosis of dyslipidemia, defined according to European Society of Cardiology (ESC) guidelines [1], while the exclusion criteria were the current involvement in other clinical trials, malignant neoplasms that could reduce life expectation, and the presence of advanced chronic kidney disease {estimated Glomerular Filtration Rate [eGFR] ≤ 15 ml/min per 1.73 m^2^, according to the Chronic Kidney Disease Epidemiology Collaboration (CKD-EPI) equation}.

All the enrolled patients signed a written informed consent and agreed to participate in a 12 month follow-up program, and the principles outlined in the Declaration of Helsinki were followed.

During the first visit, each patient underwent a detailed clinical evaluation, including medical history recording, concomitant treatments, physical examination, and assessment of anthropometric parameters and vital signs.

Data on lipid blood levels (total cholesterol, LDL cholesterol, HDL cholesterol, triglyceridemia) within the last 2 weeks were collected and compared to the targets suggested by ESC guidelines. The therapy was optimized (increasing dose and/or adding drug) in patients who did not reach the target according to the cardiovascular risk profile.

During the 3 month follow-up visit (intermediate visit) and the 12 month follow-up visit (final visit), LDL-cholesterol data were collected again, to verify if therapy optimization had allowed the achievement of suggested targets, and further therapies were prescribed to patients that had not yet reached the LDL-cholesterol targets according to ESC guidelines [1]. Side effects and compliance to drug assumption were also recorded at the intermediate and final visits.

Coming data by every ARCA cardiologist pertaining healthcare district were stored in anonymized patients’ schedules and sent to the coordinating center for statistical analysis.

### ALERT-LDL program

ARCA cardiologists were advised to closely adhere to the ALERT-LDL program, which consisted of:

– Scheduled 3 month and 12 months follow-up visits for all patients enrolled.

– Accurate reassessment of cardiovascular risk and lipidic levels at each visit.

– The well-defined and shared objective of reducing hypercholesterolaemia until to achieve guidelines suggested targets.

– Patient education sessions on the need to undergo a hypolipidemic diet and perform physical activity programs. Physicians encouraged to consume fiber, fish [1, 2], unsalted nuts [13], fruits [2, 3], and vegetables (2–3 servings per day), and to restrict the assumption of saturated fatty acids, sweets, and alcoholic beverages. Overweight and obese people were referred for a dietary consultation with a nutrition specialist. Moreover, subjects were advised to perform at least 150 min a week of physical exercise with appropriate types of activities and intensity. They were helped to set personal goals to achieve the benefits.

– Promotion of all measures that can increase patient compliance (focusing on patient knowledge of therapy’s benefits and risks, prescribing single tablet combination, checking compliance at each visit, sharing decisions).

– Availability of an e-mail service for patients who needed further information about medical treatment and lifestyle interventions during the follow-up.

### Cardiovascular risk stratification

Since new guidelines were proposed after the beginning of the study [1], the placement of each patient within a certain risk class was verified and remained unchanged from the previous evaluation [14].

The total cardiovascular risk, defined as the likelihood of a person developing a fatal cardiovascular event over the next 10 years, was calculated according to both the SCORE (Systematic Coronary Risk Estimation) system and the evaluation of clinical history. Patients with overt cardiovascular disease (i.e. chronic coronary syndrome, previous stroke, and peripheral arterial disease), diabetics with target organ damage, or patients with at least three major risk factors, (severe chronic kidney disease, familial hypercholesterolemia, or a calculated SCORE > 10%) were considered at very high-risk. Patients with markedly elevated single risk factors (in particular triglycerides > 310 mg/dL, LDL cholesterol > 190 mg/dl, or blood pressure ≥ 180/110 mmHg), patients with diabetes for more than 10 years or together with an additional risk factor, with moderate CKD or with a calculated SCORE > 5% and < 10% were considered at high risk. Diabetic patients with a disease duration less than 10 years and without other risk factors or people with a calculated SCORE between 1 and 5% were considered at moderate cardiovascular risk.

Finally, the low-risk category included all subjects with a calculated SCORE < 1% and no previous cardiovascular disease.

### Lipids’ therapeutic targets

ARCA cardiologists set cholesterol targets based on the latest guidelines [1]. In particular, the new guidelines suggest that an LDL reduction ≥ of 50% from baseline with a target < 55 mg/dl has to be achieved by the individuals at very high risk; an LDL target < 70 mg/dl can be acceptable in high-risk patients and LDL values < 100 mg/dl and < 116 mg/dl are recommended for moderate-risk and low-risk subjects, respectively.

### Statistical analysis

Categorical variables were expressed as absolute number and percentage. Continuous variables were expressed as mean ± standard deviation. The Shapiro–Wilk test was used to determine if the continuous variables were normally distributed or not. To assess statistical differences for cholesterol levels across the follow-up visit (initial vs intermediate vs final visit) ANOVA test for normally distributed variables or Friedman test for not normally distributes variables were performed. A *p* value less than 0.05 was considered statistically significant. Statistical analysis was performed using the SPSS package, version 22 (SPSS Inc., Chicago, IL).

## Results

Among the 3075 patients enrolled, 298 (9.7%) subjects were considered at low risk, 340 (11.1%) at moderate risk, 550 (17.9%) at high risk, and 1887 (61.3%) at very high risk.

The anthropometric and clinical characteristics of the study population according to cardiovascular risk stratification at baseline are summarized in Table 1. The most prevalent comorbidity was hypertension, both in the total study population (2231 patients, 72.5%) and in all the subgroups at different cardiovascular risk, followed by diabetes and chronic coronary syndrome (46.6% and 35.5% of the total population, respectively).

**Table 1:** Anthropometric and clinical characteristics of the study population

	Low (*n* = 298)	Moderate (*n* = 340)	High (*n* = 550)	Very high (*n* = 1887)	Total (*n* = 3075)
Sex Female, *n* (%)	138 (46.3)	171 (50.3)	296 (53.8)	997 (52.8)	1602 (52.1)
Age, years	64.7 ± 8.3	64.9 ± 8.5	65.2 ± 8.5	64.8 ± 8.3	64.7 ± 8.3
BMI, kg/m^2^	28.2 ± 4.7	28.3 ± 4.7	28.6 ± 4.9	28.2 ± 4.8	28.2 ± 4.8
Hypertension, *n* (%)	110 (36.9)	189 (55.6)	399 (72.5)	1533 (81.2)	2231 (72.5)
Diabetes, *n* (%)	–	26 (7.6)	336 (61.1)	1071 (56.7)	1433 (46.6)
Chronic coronary syndrome, *n* (%)	–	–	–	1092 (57.9)	1092 (35.5)
Severe CKD, *n* (%)	–	–	–	221 (11.7)	221 (7.2)
Smokers, *n* (%)	45 (15.1)	65 (19.1)	101 (18.4)	336 (17.8)	547 (17.8)

Data expressed as mean ± standard deviation or frequencies or percentage when indicated

*BMI* body mass index, *SBP* systolic blood pressure, *DBP* diastolic blood pressure, *CKD* chronic kidney disease

Table 2 shows total cholesterol, LDL and HDL cholesterol and triglyceridemia at the enrollment and after 3 and 12 months follow-up according to the different cardiovascular risk categories. After 3 months total cholesterol and LDL-c resulted reduced in all the subgroups at different cardiovascular risk (*p* < 0.05). Further intensification of therapy in patients who had not yet reached their lipid targets led to an additional reduction in LDL-c values after 12 months compared with the baseline (*p* < 0.05) and the 3 month levels (*p* = ns). Accordingly, triglycerides resulted reduced and HDL cholesterol increased in all risk categories patients at the intermediate and final visit in respect to baseline (*p* < 0.05).

**Table 2:** Lipid profile at baseline, intermediate (after 3 months) and final visit (after 12 months)

	Low (*n* = 298)	Moderate (*n* = 340)	High (*n* = 550)	Very high (*n* = 1887)	Total (*n* = 3075)	p*	p**	p***
Tot-cholesterol								
- Baseline	191.2 ± 40.5	193.7 ± 39.6	204.3 ± 41.4	206.2 ± 40.3	199.5 ± 40.3	< 0.05	< 0.05	ns
- Intermediate visit	168.3 ± 31.0	171.6 ± 30.7	152.9 ± 31.1	154.7 ± 30.6	158.5 ± 30.7			
- Final visit	145.1 ± 35.6	149.5 ± 33.3	147.3 ± 30.8	142.6 ± 32.9	144 ± 47.8			
LDL cholesterol								
- Baseline	122.7 ± 35.3	126.5 ± 35.5	131.8 ± 37.1	141.5 ± 35.3	135 ± 37.0	< 0.05	< 0.05	ns
- Intermediate visit	95.5 ± 41.8	98.1 ± 41.9	80.7 ± 26.6	81.9 ± 26.3	85 ± 39.2			
- Final visit	75.5 ± 26.5	78.3 ± 32.6	73.3 ± 24.6	63.4 ± 18.9	67.6 ± 26.3			
HDL cholesterol								
- Baseline	47.5 ± 12.4	48.4 ± 12.2	49.5 ± 12.4	45.7 ± 12.2	47.2 ± 12.2	< 0.05	< 0.05	ns
- Intermediate visit	51.6 ± 12.2	49.5 ± 12.5	51.5 ± 12.9	47.4 ± 12.6	49.5 ± 12.6			
- Final visit	53.1 ± 11.8	49.9 ± 12.0	52.2 ± 11.5	48.8 ± 13.0	50.1 ± 12.0			
Triglycerides								
- Baseline	145.2 ± 65.1	147.3 ± 62.6	150.4 ± 66.7	152.0 ± 69.8	148.5 ± 69.8	< 0.05	< 0.05	ns
- Intermediate visit	118.2 ± 41.2	121.5 ± 41.9	120.1 ± 43.7	122.9 ± 41.3	120.2 ± 41.7			
- Final visit	106 ± 33.1	114.3 ± 40.2	112.5 ± 35.6	106.8 ± 40.1	110.7 ± 38.9			

Data expressed as mean ± standard deviation

p*Comparison between baseline and intermediate data in the study population and in subgroups at different cardiovascular risk

p**Comparison between baseline and final data in the study population and in subgroups at different cardiovascular risk

p***Comparison between intermediate and final data in the study population and in subgroups at different cardiovascular ris

**Fig. 1:**
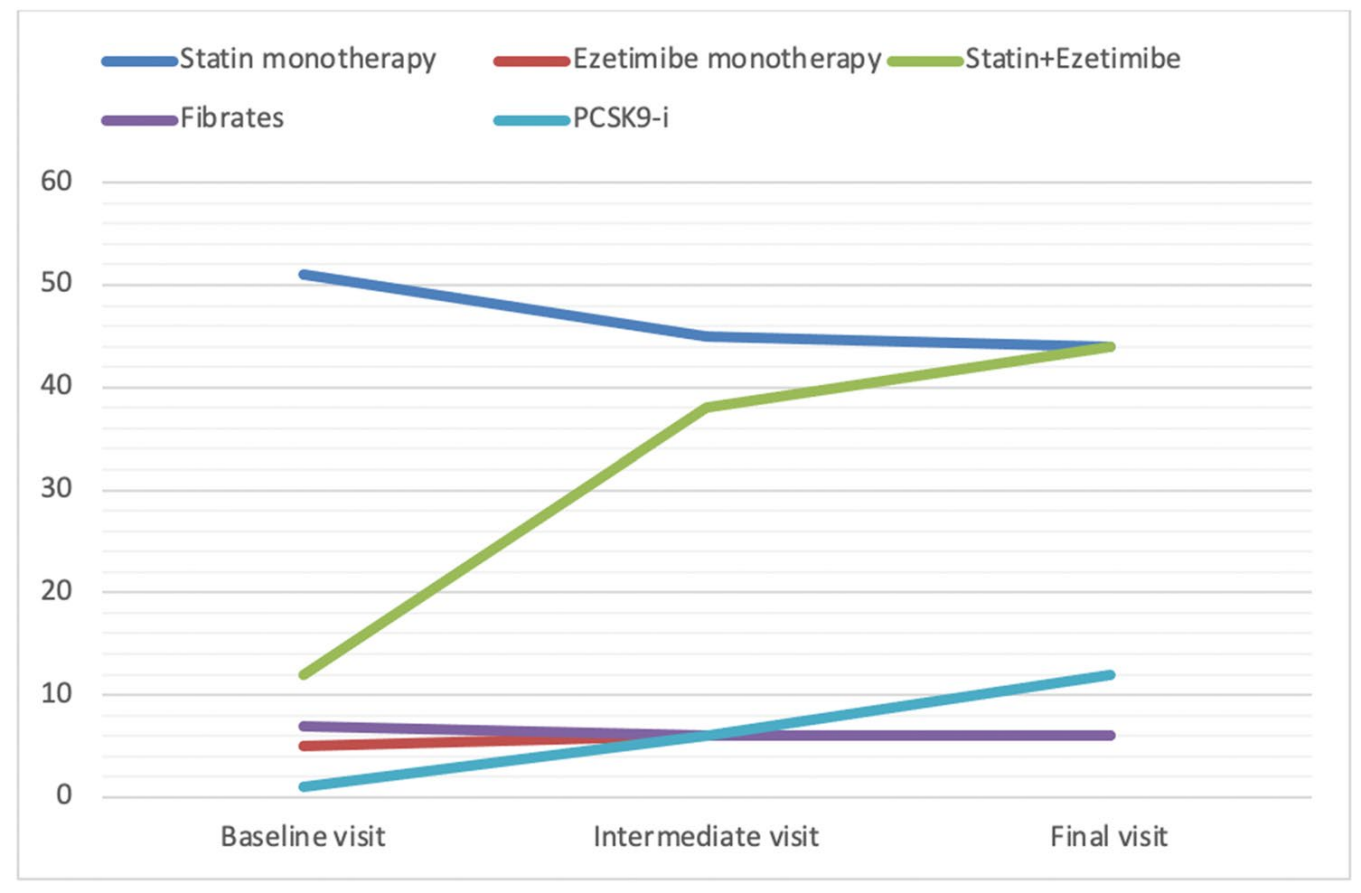
Hypolipidemic drugs in the study population at the baseline, intermediate (after 3 months) and final (after 12 months) visit

Table 3 and Fig. 1 report hypolipidemic therapies at the enrollment and after 3 and 12 months follow-up for the different risk categories. Patients assuming statins, both in monotherapy and in combination with ezetimibe, increased during the follow-up (63% at the enrollment vs 89% 1 year later, *p* < 0.05). At the beginning of the study 55.0% of them were treated with high-intensity statins (atorvastatin 36.9% and rosuvastatin 18.1%) and 45.0% with moderate-intensity statins (simvastatin 40.3%, pravastatin 3.7%, lovastatin 1.0%). At the end of the study, the use of high-intensity statins increased from 55 to 71.2%, and moderate-intensity statins treatment reduced from 45 to 28.8%. The percentage of patients assuming Nutraceuticals, Fibrates and Omega 3 remained essentially unchanged during the follow-up. At the enrollment, only 42 patients at very high risk (2.2% of the total very high-risk population) were treated with PCSK-9 inhibitors, whereas, at the end of the study the percentage increased both in high (5.8%) and very high-risk patients (18.4%, *p* < 0.05).

**Table 3:** Hypolipidemic drugs prescribed at the baseline, intermediate (after 3 months) and final visit (after 12 months)

	Low (*n* = 298)	Moderate (*n* = 340)	High (*n* = 550)	Very high (*n* = 1887)	Total (*n* = 3075)
Statin monotherapy, *n* (%)					
- Baseline	116 (38.9)	178 (52.4)	217 (39.5)	1060 (56.1)	1571 (51.1)
- Intermediate visit	203 (68.1)	213 (62.7)	341 (62.0)	614 (32.6)	1371 (44.6)
- Final visit	215 (72.1)	255 (75.0)	401 (72.9)	528 (27.9)	1399 (45.5)
Ezetimibe monotherapy, *n* (%)					
- Baseline	–	37 (10.9)	28 (5.1)	105 (5.7)	170 (5.5)
- Intermediate visit	15 (5.0)	41 (12.1)	42 (7.6)	84 (4.6)	182 (5.9)
- Final visit	18 (6.0)	40 (11.8)	47 (8.5)	81 (4.3)	186 (6.0)
Statin + ezetimibe, *n* (%)					
- Baseline	39 (13.0)	5 (1.5)	21 (3.8)	297 (15.7)	362 (11.8)
- Intermediate visit	42 (14.0)	21 (6.2)	69 (12.5)	1025 (54.3)	1157 (37.6)
- Final visit	63 (21.1)	38 (11.2)	91 (16.5)	1151 (61.0)	1343 (43.7)
Nutraceuticals, *n* (%)					
- Baseline	8 (2.7)	46 (13.5)	35 (6.4)	64 (3.4)	153 (5.0)
- Intermediate visit	10 (3.4)	64 (18.8)	38 (6.9)	42 (2.2)	154 (5.0)
- Final visit	10 (3.4)	60 (17.7)	38 (6.9)	33 (1.75)	141 (4.6)
Fibrates, *n* (%)					
- Baseline	31 (10.4)	11 (3.2)	23 (4.2)	148 (7.8)	213 (6.9)
- Intermediate visit	16 (5.4)	8 (2.4)	25 (4.5)	140 (7.4)	189 (6.1)
- Final visit	15 (5.0)	8 (2.4)	28 (5.1)	131 (6.9)	182 (5.9)
Omega 3, *n* (%)					
- Baseline	13 (4.4)	21 (6.2)	18 (3.3)	61 (3.2)	113 (3.7)
- Intermediate visit	53 (17.8)	52 (15.3)	31 (5.6)	96 (5.1)	232 (7.5)
- Final visit	58 (19.4)	55 (16.2)	33 (6.0)	94 (4.9)	240 (7.8)
PCSK-9 inhibitors, *n* (%)					
- Baseline	–	–	–	42 (2.2)	42 (1.4)
- Intermediate visit	–	–	21 (3.8)	169 (8.9)	190 (5.5)
- Final visit	–	–	32 (5.8)	348 (18.4)	380 (12.4)

**Fig. 2:**
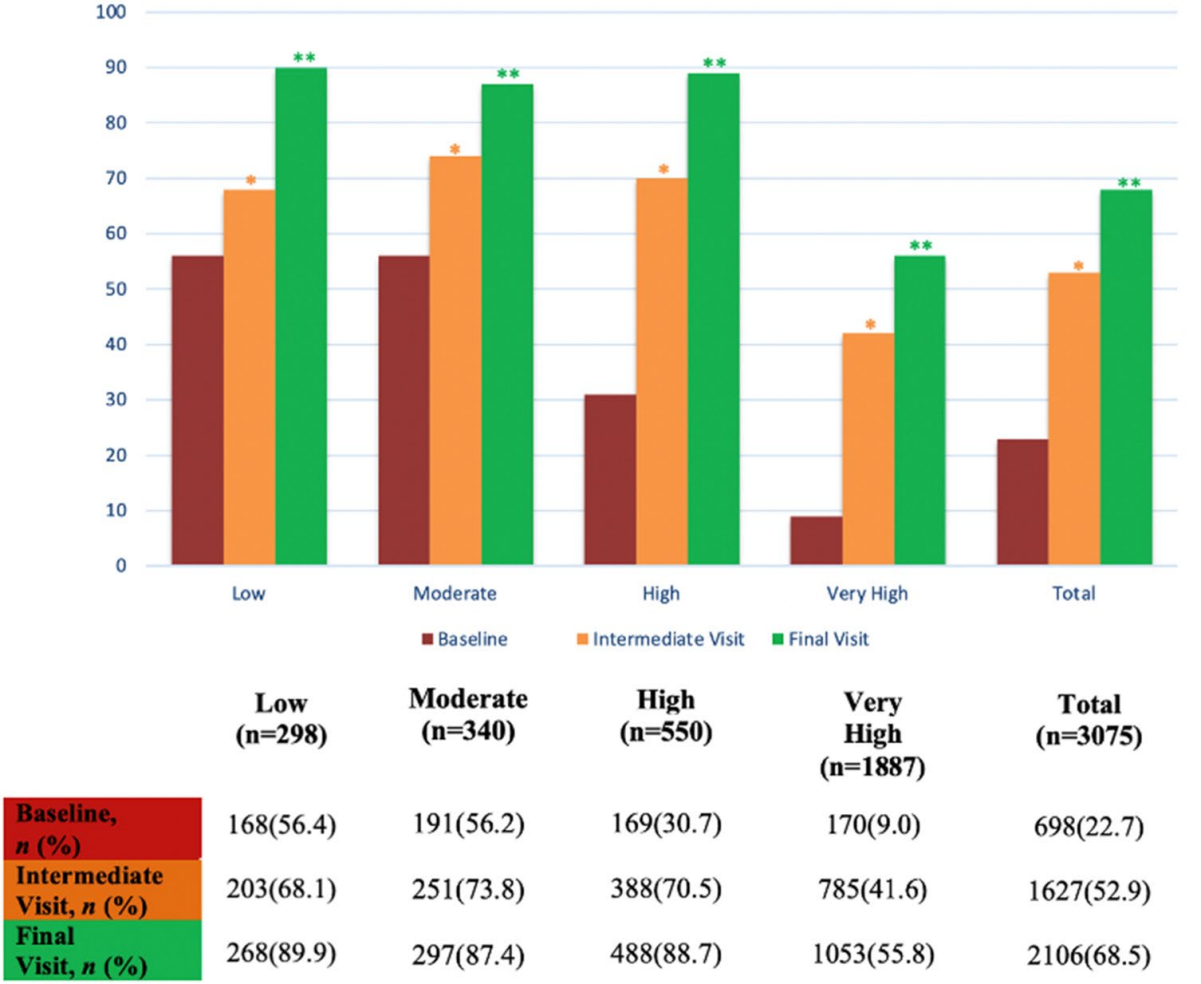
Patients at different cardiovascular risk on target at the baseline, intermediate (after 3 months) and final visit (after 12 months).*Comparison between intermediate visit and baseline in the study population and in subgroups at different cardiovascular risk, *p* < 0,05.**Comparison between final visit and baseline in the study population and in subgroups at different cardiovascular risk, *p* < 0,01

Figure 2 shows the percentage of patients that reached the lipid target at the beginning of the study and after 3 and 12 months follow-up. At the beginning, only 698 patients (22.7%) showed cholesterol levels comprised in the guidelines’ suggested referring values. The percentage was even lower in very high-risk patients (170 patients, 9.0% of the 1887 patients at very high risk). In this subgroup, the number of patients who reached the target increased after 3 (41.6%, *p* < 0.05 versus patients on target at baseline) and 12 months (55.8%, *p* < 0.01 vs baseline) follow-up. This trend was observed in all risk categories resulting in a significant increase in the percentage of patients on target after 3 months (52.9%) and at the end of the study (68.5% of the study population, *p* < 0.01 vs patients on target at the enrollment).

Major cardiovascular events (non-fatal myocardial infarction, stroke, other ischemic events) occurred in 29 patients at high and very high risk (0,94% of the total population) (Table 4). Among these, five high-risk patients at the enrollment experienced a major cardiovascular event during the follow-up [2, 3] and were therefore considered at very high risk. Nine low-risk patients at the beginning developed diabetes and were classified as moderate-risk subjects. All these patients did not reach the lipid targets suggested for their risk category either at the beginning of the study or at the follow-up visits.

**Table 4:** Cardiovascular events in high and very high-risk patients and in study population

	High (*n* = 550)	Very high (*n* = 1887)	High + very high (*n* = 2437)	Total (*n* = 3075)
Major cardiovascular events, *n* (%)	5 (0.90)	24 (1.27)	29 (1.24)	29 (0.94)
Non-fatal myocardial infarction, *n* (%)	3 (0.54)	10 (0.53)	13 (0.53)	13 (0.42)
Non-fatal stroke, *n* (%)	2 (0.36)	12 (0.64)	15 (0.61)	15 (0.49)
Other non-fatal ischemic events, *n* (%)	0	2 (0.1)	2 (0.08)	2 (0.06)

Multivariate analysis among patient's characteristics (sex, age, BMI) and comorbidities (hypertension, CKD, diabetes, chronic coronary syndrome) and the achievement of lipid target for each CV risk category were performed. None of the variables resulted associated with the achievement of lipid target for each CV group.

## Discussion

Dyslipidaemia is one of the main determinants of cardiovascular burden and guidelines recommend very stringent LDL-c targets particularly in high-risk patients [1]. In real-life clinical practice the achievement of these therapeutic objectives may be hampered by different causes such as therapeutic inertia, long waiting time between follow-up visits, poor patient compliance, low lipid measurement rates, occurrence of adverse events [15].

ALERT-LDL study has been carried out to detect the adherence to guidelines-oriented dyslipidaemia’s treatment and evaluate the efficacy of a lipid values management program launched by 12 ARCA cardiologists of the territorial healthcare districts.

Only 22.7% of the patients were on target at the enrollment, mainly due to the low percentage of very high-risk patients (only 9% on target at the initial visit). An improvement of the lipidic profile was already observed at the intermediate visit, with a significant increase in the percentage of very high-risk patients on target (41.6%). At the intermediate visit, subsequent efficacy measures further lowered LDL-c levels, with a significantly higher percentage of patients who reached the targets at the end of the follow-up in all group. Accordingly, total cholesterol resulted reduced and HDL cholesterol increased at the intermediate and final visit, and triglycerides levels also showed a significant reduction during the follow-up.

Poor adherence or discontinuation of therapy due to statin intolerance has been reported as one of the main causes of failure to reach the lipid target [16]. ARCA cardiologists investigated whether there was a real diagnosis of statin intolerance, particularly with regard to muscular symptoms (myalgia, muscle stiffness and tenderness, cramps and loss of muscle strength). Changes in the dose (statin dechallenge) or switch with other statins were suggested to reduce the occurrence of intolerance and, consequently, to improve the effectiveness of hypolipidemic therapy. Thus, the percentage of patients diagnosed with true complete statin intolerance reduced from 16 to 6%.

Changes in lipid balance were achieved by combining drug therapies (dosage adjustments in current therapies and addition of new hypolipidemic drugs) and non-pharmacological interventions. The main therapeutic modifications regarded the add-on of a single tablet statin-ezetimibe combination, which was preferred by physicians in respect to multiple pills as a method to improve adherence [17, 18]. A single tablet statin-ezetimibe combination was taken by 11.8% of hypercholesterolemic patients at the beginning with an increase to 43.7% at the end of the follow-up. Likewise, the percentage of patients assuming PCSK-9 inhibitors increased from 1.4 to 12.4% at the end of the study, mostly prescribed in subjects at very high risk not achieving the required target with the maximum tolerated dose of a statin and ezetimibe (from 2.2% at the initial visit to 18.4% at the end of the follow-up). The high and very high-risk patients well tolerated PCSK-9 inhibitors and showed elevated compliance to the therapy, according to the reference randomized trials reporting a marked tolerance and adherence to the treatment with the consequent cardiovascular benefits [19–25]. Non-pharmacological interventions included tailored dietary regimen, planned physical exercise, frequent interactions with the cardiologist and checks of cholesterol levels, patient information and education, e-mail service.

The main findings of our study suggest that the stringent lipid targets recommended by the scientific societies in real-life are achieved by a rather low percentage of patients [26]. A relatively modest number of high or very high-risk patients reaching the LDL-c targets has also been observed in previous studies carried out on large cohorts of outpatients in Italy and other countries [27–29]. However, the promotion and sharing of specific initiatives, emphasizing the effectiveness of cholesterol control and the crucial importance of periodic follow-up, carried out by the referring cardiologists of the pertaining healthcare districts, increase the percentage of patients on target [13, 30–32]. In our study the improvement in lipid profile was obtained within a few months thanks to the careful program adopted regarding diet and physical activity, the periodic follow-up, the frequent recurrence of cholesterol dosages, the correct use of risk stratification, the periodic evaluation of the target’s achievement and update of the therapies. Finally, these results were also promoted by the opportunity that ARCA cardiologists had to prescribe by themselves the novel, powerful PCSK-9 inhibitors.

ALERT-LDL study suggests that clinicians should assess cardiovascular risk and define precise LDL-c goals at each visit to prescribe the most beneficial therapy early, reducing the time of exposure to high cholesterol levels, which is considered one of the most important components of the cardiovascular risk [5]. Lipid-lowering therapies can reduce the occurrence of cardiovascular events and exert greater health and cost-effectiveness benefits among high-risk people [31]. Thus, public health programs aimed to achieve tailored therapeutic objectives should be aggressively implemented.

### Limitations

The ALERT-LDL registry accurately describes the characteristics of a real-world population of patients consecutively referring to the healthcare territorial districts. The majority of the patients fall into the very high-risk category, limiting the general applicability of the results, but these are the patients who most need to reach the guideline's suggested targets. Moreover, the lack of patients with low to moderate risk might not be due to the characteristics of the target population but related to poor awareness of their increased cardiovascular risk. Therefore, the results of our study suggest that different strategies (f.e. social media) are likely needed to improve patients’ awareness and the approach referral to outpatient cardiologists to manage their cardiovascular risk.

## Conclusion

Only one-fifth of dyslipidaemic outpatients referring to the pertaining healthcare districts were on target according to their cardiovascular risk, most of them belonging to the very high-risk group. When included in territorial programs carried on by their cardiologists and specifically aimed at the control of the lipid profile, the percentage of patients on target increased in all risk categories. Thus, the results of this study suggest carefully implementing measures that encourage physicians and patients to achieve the correct lipid profile according to the global cardiovascular risk.

## Data Availability

The datasets used and/or analyzed during the current study are available from the corresponding author on reasonable request.
